# Global expression pattern of genes containing positively selected sites in European anchovy (*Engraulis encrasicolus* L.) may shed light on teleost reproduction

**DOI:** 10.1371/journal.pone.0289940

**Published:** 2023-08-11

**Authors:** Vahap Eldem, Gökmen Zararsız, Melike Erkan

**Affiliations:** 1 Faculty of Sciences, Department of Biology, Istanbul University, Istanbul, Turkey; 2 Department of Biostatistics, Erciyes University, Kayseri, Turkey; Universiti Malaysia Terengganu, MALAYSIA

## Abstract

European anchovy is a multiple-spawning and highly fecundate pelagic fish with high economic and ecological significance. Although fecundity is influenced by nutrition, temperature and weight of spawners, high reproductive capacity is related to molecular processes in the ovary. The ovary is an essential and complex reproductive organ composed of various somatic and germ cells, which interact to facilitate the development of the ovary and functional oocytes. Revealing the ovarian transcriptome profile of highly fecundate fishes provides insights into oocyte production in teleosts. Here we use a comprehensive tissue-specific RNA sequencing which yielded 102.3 billion clean bases to analyze the transcriptional profiles of the ovary compared with other organs (liver, kidney, ovary, testis, fin, cauda and gill) and juvenile tissues of European anchovy. We conducted a comparative transcriptome and positive selection analysis of seven teleost species with varying fecundity rates to identify genes potentially involved in oogenesis and oocyte development. Of the 2,272 single copies of orthologous genes found, up to 535 genes were under positive selection in European anchovy and these genes are associated with a wide spectrum of cellular and molecular functions, with enrichments such as RNA methylation and modification, ribosome biogenesis, DNA repair, cell cycle processing and peptide/amide biosynthesis. Of the 535 positively selected genes, 55 were upregulated, and 45 were downregulated in the ovary, most of which were related to RNA and DNA transferase, developmental transcription factors, protein kinases and replication factors. Overall, our analysis of the transcriptome level in the ovarian tissue of a teleost will provide further insights into molecular processes and deepen our genetic understanding of egg production in highly fecund fish.

## Introduction

The ecological sustainability of marine ecosystems is closely related to the reproductive periods, fecundity and growth patterns of small fishes (e.g. anchovy, sardine and round herring), which play a key role in the food chain and energy flow. Anchovies (*Engraulis* sp.) are small pelagic fishes abundant and ubiquitous in the epipelagic zone of the world’s oceans and are a key component of marine ecosystem due to their high biomass productivity. Among anchovies, the European anchovy (*Engraulis encrasicolus* L., 1758) is one of the most important fish species in both the economical and ecological senses, and is widely distributed along the Eastern Atlantic coastline from the North Sea to central Africa, and the Mediterranean, Black and Azov Seas [1–3]. According to FAO (2019) estimates, global capture production of anchovies (combining data from 3 species: *E*. *ringers*, *E*. *japonicus* and *E*. *encrasicolus*) was estimated at about 7 million tones per year between 2015 and 2019 (https://www.fao.org/fishery/en/statistics). From an ecological standpoint, large schools of anchovies predominantly occupy the mid-trophic level of the food chain, transferring energy and nutrients from zooplankton (even phytoplankton) to higher trophic levels [4–6]. High-productive capacity (i.e., excessive egg production), early maturation and feeding on a wide range of prey may contribute to the apparent ecological success of anchovies despite their short lifespan.

Oogenesis or egg production follows a universal pattern in fishes, but most species show fluctuations in spawning frequency, fecundity and egg size [7]. As a reproductive parameter, fecundity (also known as, total eggs produced per female in a season) is a useful indicator of reproductive success. Although most marine fishes, particularly pelagic species, are generally considered to be highly fecund, fishes exhibit considerable variation in terms of egg numbers. Probably the most striking example of high fecundity was seen in the ocean sunfish (*Mola mola*, Molidae), for which approximately 300 million oocytes were estimated in a single female at the time of examination [8,9]. Another intriguing example is the large dolphinfish (*Coryphaena hippurus*, Coryphaenidae), which can produce more than 100 million eggs per year [7]. In general, the highest fecundity in pelagic spawners is found in Atlantic cod (*Gadus morhua*) with numbers ranging from thousands to several million, as well as small fishes such as anchovies. As an iteroparous spawner, each individual anchovy (*E*. *encrasicolus*) can typically spawn multiple times per year and more than 500,000 eggs may be laid in total (https://www.fishbase.se/summary/66) with a comparatively long reproductive season. A study on the reproductive biology and fecundity of the anchovy (*E*. *encrasicolus*) in the Bay of Biscay conducted between 1987 and 1992, found that the total reproductive output of this species could range from approximately 9,000 to 11,000 eggs per unit gonad-free body weight (g). Additionally, the fecundity of the Bay of Biscay anchovy was found to vary from 110,000 eggs (for a 10 g female) to 350,000 eggs (for a 40 g female) over a 2.5-month spawning season [10]. In another study, the batch fecundity (i.e., the number of eggs released per fish during a single spawning event) of *E*. *encrasicolus* was found to range from 981 to 21,750 eggs (11,998 ± 5397, *n* = 31) using the oocyte size-frequency method to quantify the cohort of oocytes. Batch fecundity was found to be positively correlated with the size of the specimen [11]. Although the fecundity of anchovy fish in the Black Sea is not currently recorded in the FishBase database, a study by Lisovenko and Andrianov found that the total number of eggs spawned by one average female anchovy during the spawning season ranges from 138,203 to 231,184 [12]. On average, each female anchovy spawns more than 50 times per year [12]. The fecundity of anchovy from different areas may vary due to a combination of environmental factors (such as water temperature) and genetic differences (such as body size) among different populations [13]. All these studies indicate that *E*. *encrasicolus* is a multiple-spawning and highly fecundate pelagic fish. In contrast, even much smaller species, such as zebrafish *(Danio rerio*) or three-spined stickleback (*Gasterosteus aculeatus*) produce only several hundred eggs (https://www.fishbase.se/).

Studying the cellular processes of ovaries and testis at the transcriptome level in highly fecundate fish is a straightforward way to explore teleost reproduction and development. Detection of gene expression levels and molecular networks in gonadal tissues—in other words, illuminating molecular processes specific to these tissues—is essential to understanding the reproductive biology of teleosts [14–16]. In particular, the ovary is known to be one of the most active organs for cell division, differentiation and maturation in an adult fish body [17–20]. The ovarian transcriptome profile and dynamics are directly linked to egg production capacity; however, little is known about the molecular processes of high-reproduction capacity in fishes. RNA-seq or whole-transcriptome profiling is emerging as a preferred strategy for non-model organisms and this approach provides detailed information on the expression level of protein-coding and non-coding genes [21,22]. Tissue-specific transcriptome profiling is considered a first step for elucidating the biological process of target organs such as ovaries. Moreover, this methodology allows for the detailed characterization of ovarian development, regulation of multiple signaling pathways and identifiying hub genes for oocyte maturation and ovulation [23–26].

Here, we present a large scale multi-tissue (ovary, testis, kidney, liver, gill, fin and caudal tissues) and juvenile transcriptome for *E*. *encrasicolus* using RNA-Seq. For comparision, we also include our previous data on muscle gene expression in *E*. *encrasicolus* using a similar RNA-Seq approach [27]. We characterize the genome-wide ovarian gene expression profile and gene expression network in *E*. *encrasicolus* by tissue-specific expression analysis. In addition to providing transcriptomic data and tissue-specific expression profiles, identifying positively selected sites in protein coding genes by comparative evolutionary analysis with other fish species provides a good starting point for understanding egg production in highly fecundate fishes. Taken together, this data allows us to supply valuable transcriptomic resources for the reproductive biology of clupeid fishes and shed light on potential genetic clues to the reproductive success of highly fecund fishes.

## Methods

### Ethical statement

The fish specimens were acquired in a deceased state from local fishermen in the fish market, therefore ethical aspects are not applicable.

### Collection of specimens

Adult female and male anchovies were acquired from fishermen at fish catching sites of Black Sea region (41°14’41.1’’ N; 29°20’40.1’’ E), making ethical concerns unapplicable. The length of adult anchovies varied between 11.0 and 15.0 cm with an average of 12.80 cm. Juvenile anchovies (3.5 cm in length on average) were kindly provided by Prof. A.C. Gücü (Middle East Technical University) from the project titled “*Determination of the Black Sea anchovy stocks by Acoustic Model and Generating Continuous Monitoring Model for the National Fisheries Data Collection Program*”. The collected juveniles and dissected tissues were immediately immersed in liquid nitrogen and stored in a −80°C freezer until RNA extraction. A total of 26 individuals were subjected to *de novo* transcriptome analysis, encompassing various tissue types: Ovary (3 samples), Testis (3 samples), Cauda (3 samples), Gill (3 samples), Fin (3 samples), Juvenile (5 samples), Muscle (2 samples), Liver (2 samples), and Kidney (2 samples).

### RNA-Seq library preparation and sequencing

Seven tissue types (liver, kidney, ovary, testis, fin, cauda, gill) were dissected and used for total RNA isolation. For juvenile anchovy samples, only total tissue mass including connective tissues and reproductive organs was obtained for total RNA extraction due to their small size and mucus-like nature. Total RNAs were extracted from dissected tissues using TRIzol^®^ reagent (Invitrogen) according to standard protocol. Concentrations of RNA samples were measured with a Nanodrop^©^ 2000c (Thermo Scientific). The integrity of the RNA (RIN) was determined using an Agilent 2100 Bioanalyzer (Agilent Technologies) and only RNA samples with RIN number >8.0 were further processed. RNA-Seq library construction and sequencing were carried out by the Genetic Engineering and Biotechnology Institute (GEBI) of TUBITAK using standard protocols and sequenced on the Illumina HiSeq 2000 with paired-end 100 bp reads. The RNA-Seq libraries for gill, fin and caudal tissues were sequenced on a DNBSEQ-400 instrument (MGI) with paired-end 100 bp reads. Raw transcriptome sequences can be found under BioProject PRJNA348159 and PRJNA896144. Transcriptome data from muscle tissues was obtained from our previous work [27]. In the present study, a subset of tissue samples, including ovary, testis, juvenile, kidney, liver, and muscle, were subjected to sequencing using the Illumina HiSeq2000 platform. In contrast, gill, cauda, and fin tissues were sequenced using the MGI DNB-SEQ 400 platform (S1 Table). However, it is noteworthy that potential cross-platform variation is anticipated to be minimal, as both platforms exhibit comparable characteristics regarding read lengths, error rates, and sequencing depths (S3 File).

### Data processing and *de novo* transcriptome assembly

Read quality of raw reads was assessed using the FastQC v0.11.9 (https://github.com/s-andrews/FastQC) and MultiQC v1.13 tools [28]. Prior to *de novo* transcriptome assembly, raw reads were trimmed and quality-filtered with fastp v0.22.0 [29] using the following criteria: (i) removing adapter sequences, (ii) discarding the low-quality reads (Phred quality score less than 20, Q ≥ 20) and ambiguous nucleotides (‘N’ at the end of reads), and (iii) removing the short read length sequences (less than 50 bp). High-quality sequence reads were assembled using Trinity v2.13.0 [30] with a default k-mer size of 25 and min_contig_length = 300. *De novo* assembled contig sequences were clustered following Corset v1.09 [31] based on shared reads. The gVolantes web server was used for assessing the completeness of assembled transcriptomic data using Benchmarking Universal Single-Copy Orthologs (BUSCO v5) and the Actinopterygii dataset [32]. Next, we used the online server DOGMA to assess transcriptome integrity using conserved protein domains and domain assembly information [33].

### Identification of differentially expressed genes

Because *E*. *encrasicolus* has no reference genome, the estimation of transcript abundance from each sample was performed using RSEM v1.3.3 (RNA-Seq by Expectation-Maximization) under default settings [34]. Initially, high-quality reads for all samples were aligned to the reference transcriptome assembly using Bowtie v2.4.0 [35], then the abundance of each transcript was estimated using RSEM. Prior to DEG analysis, transcript isoforms exhibiting weak expression or lower expression were removed using the "*filter_low_expr_transcripts*.*pl*" script (https://github.com/trinityrnaseq/trinityrnaseq/tree/master/util) utilizing the *"—highest_iso_only*" parameter to retain only the most abundantly expressed isoform per gene. We performed the isoform-level expression profiling per gene in reproductive and somatic tissues. The differentially expressed genes (DEGs) between tissues were identified by Bioconductor package DESeq2 v1.22.2 [36]. The DEGs with a |log_2_(Fold Change)| > 1.5 and a false discovery rate of ≤ 0.001 were considered statistically significant. Differential expression analysis was performed on reproductive tissues (ovary, testis) versus somatic tissues (cauda, gill, fin, muscle, liver, kidney) to gain insight into potential genes involved in teleost reproduction.

### Functional annotation and enrichment analysis

The *de novo* assembled transcripts of *E*. *encrasicolus* were functionally annotated using Trinotate pipeline v3.2.0 [37] with a cutoff e-value of 1e-10. First, contig sequences (>300 bp) were scanned with Transdecoder v.5.0.2 (http://transdecoder.github.io). The coding CDS ("longest_orfs.cds") and peptide sequences ("longest_orfs.pep") were predicted using the "TransDecoder.Predict" script. The "*single_best_only*" option was supplied to retain only the best-scoring open reading frame (ORF) for each transcript. At the prediction stage, the option "-retain (blastp|pfam) hits" was utilized to reduce the number of false-positive ORF (open reading frame) discoveries. The contig sequences were scanned by BlastX v2.11.0 against UniProt/Swiss-Prot (release 2022_04) and predicted ORFs were analyzed with BlastP v2.11.0 against the NCBI NR (non-redundant genes) database to discard potential contaminant sequences. After filtering non-vertebrate sequences, filtered contig sequences were analysed by BlastX and the corresponding ORFs scanned by BlastP v2.11.0 against the UniProt/Swiss-Prot database. ORFs were searched with (i) hmmscan v 3.3.2 [http://hmmer.org/] against Pfam-A release 35.0, (ii) TmHMM v.2.0, and (iii) SignalP v6.0. RNAmmer was used to scan the contig sequences for predicting rRNA genes [38]. The results of these analyses were loaded into a locally established database and merged using Trinotate. The online webtool TRAPID v2.0 was used to functionally annotate filtered contig sequences, which compared anchovy transcripts against eggNOG 5.0 database with an e-value threshold of 10^−5^ for significant similarity search [39]. Using Ensemble Protein ID information from orthologous zebrafish, Gene Ontology (GO) and KEGG pathway functional enrichment analysis of differentially expressed genes (DEGs) was performed using TRAPID v2.0 [39] and ShinyGO [40]. Functional enrichment of DEGs was assessed using Fisher’s exact test, with terms showing >10% enrichment at an FDR-adjusted *P* value of <0.05 marked as enriched [41].

### Orthologs identification and detecting positively selected genes

To determine the coding region in the assembled transcriptome, we used GeneMarkS-T [42] with default parameters. The algorithm of GeneMarkS-T is estimated by unsupervised training, which makes manually curated preparation of training sets unnecessary. This unsupervised training is robust in terms of transcript assembly errors. To improve the accuracy of protein-coding gene predictions, we compared the sequences predicted by GeneMarkS-T to those obtained from the homology-based Transdecoder analysis. We then continued the orthologous analysis using the common coding sequences of both methods. For orthologous gene identification, the reference proteomes of *Danio rerio*, *Gasterosteus aculeatus*, *Oreochromis niloticus*, *Takifugu rubripes*, *Tetraodon nigroviridis* and *Xiphophorus maculatus* were collected from Ensemble (release 104). We kept the longest protein sequence for each gene and performed an all-against-all using BlastP v2.11.0 with an e-value threshold of 10^−10^) [43]. A gene tree was constructed for each group using Treefam [44]. Using reciprocal Best Hits (RBH) methodology, we obtained *E*. *encrasicolus*’ single copy gene orthologs according to zebrafish single copy genes. All orthologous coding regions of these seven species were aligned by PRANK [45] using the GUIDANCE [46] pipeline. PRANK is a probabilistic multiple alignment program for DNA, codon and amino-acid sequences. The standard PRANK algorithm is based on an exhaustive search of the best pairwise solution; the GUIDANCE assigns a confidence score for each residue, column and sequence in a multi alignment from Prank, so guidance can be used for weighting, filtering or masking unreliably aligned positions in sequence alignments before positive selection using the branch-site *dN/dS* test. PhyML v3.0 [47] was employed to infer the phylogeny of the seven fishes based on protein residues translated from multi alignments of single copy orthologs. We then used the phylogeny and multi alignment to detect positive selection using the branch-site model with the CodeML program of the PAML v4.0 (phylogenetic analysis using maximum likelihood) package (v4.6; settings: model = 2, NS sites = 2) [48]. The branch-site test for positive selection assumes that the branches in the phylogenetic tree can be divided into foreground branches (where codons are under positive selection) and background branches (without positive selection). Model A1 (H0; CodeML settings: fix_omega = 1, omega = 1), where a codon may evolve neutrally or under purifying selection, was compared with model A, where codons on the branch of interest can be under positive selection (HA; CodeML settings: fix_omega = 0, omega = 1). Likelihood ratio test *P*-values were computed assuming that the null distribution was a *χ2*-distribution with 1 degree of freedom and adjusted for multiple testing with a FDR threshold of 0.05 [49]. Afterwards, we removed continuous sites, which may be produced by incorrect gene prediction and assembly, and also manually checked the most statistically significant genes. The predicted protein-protein interaction (PPI) network of top PSGs was constructed using the Search Tool for the Retrieval of Interacting Genes (STRING; https://string-db.org/) database. The hub genes were then screened and only classes with FDR < 0.05 for functional enrichment were selected for highlights (PPI enrichment p-value:0.000135).

## Results

### A survey of fish fecundity through FishBase database screening

**Fig 1:**
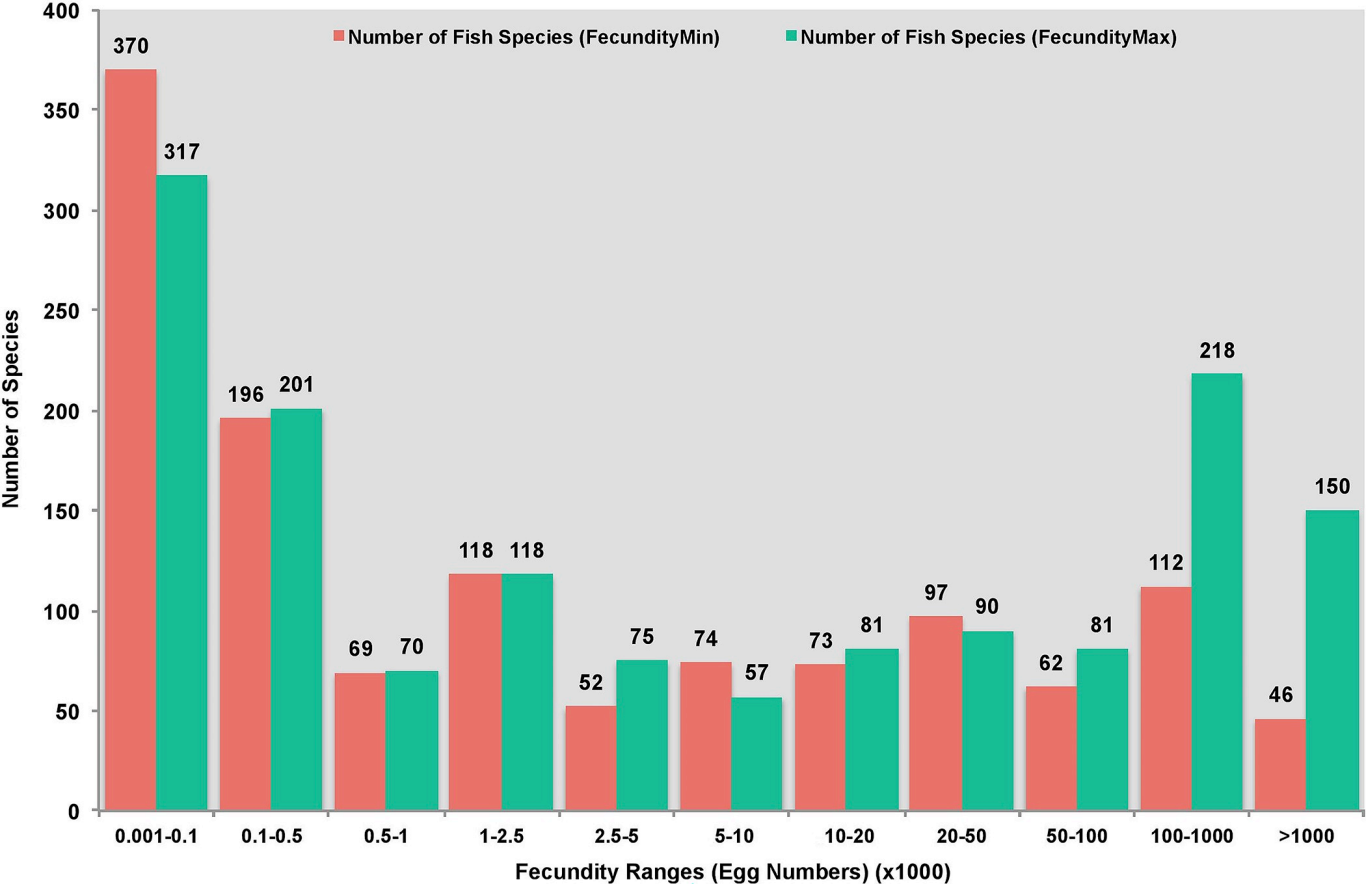
The fecundity ranges (FecundityMin and FecundityMax) of ray-finned fish species aggregated into eleven categories from 0.001 to 1000 (total number of oocytes).

The minimum and maximum fecundity values (egg numbers) for marine fish species were collected from FishBase using the R package rfishbase [50]. Contrary to the general understanding that fishes are highly fecund vertebrates, the database search showed significant fluctuation in their fecundity patterns (Fig 1).

As seen in Fig 1, the fecundity values or the total number of oocytes, ranging from ~10^2^ to >10^6^, were partitioned into eleven groups. The fishes of genus *Engraulis* fall under the 100–1000 range alongside other highly fecund species such as *Gadus chalcogrammus*, *Clupea harengus*, *Sardina pilchardus* and *G*. *morhua*. These results largely align with the previous fecundity study carried out with *E*. *encrasicolus* in the Black Sea (egg numbers per female anchovy ranging from 138,203 to 231,184) [12].

### Sequencing and *de novo* transcriptome assembly

**Fig 2:**
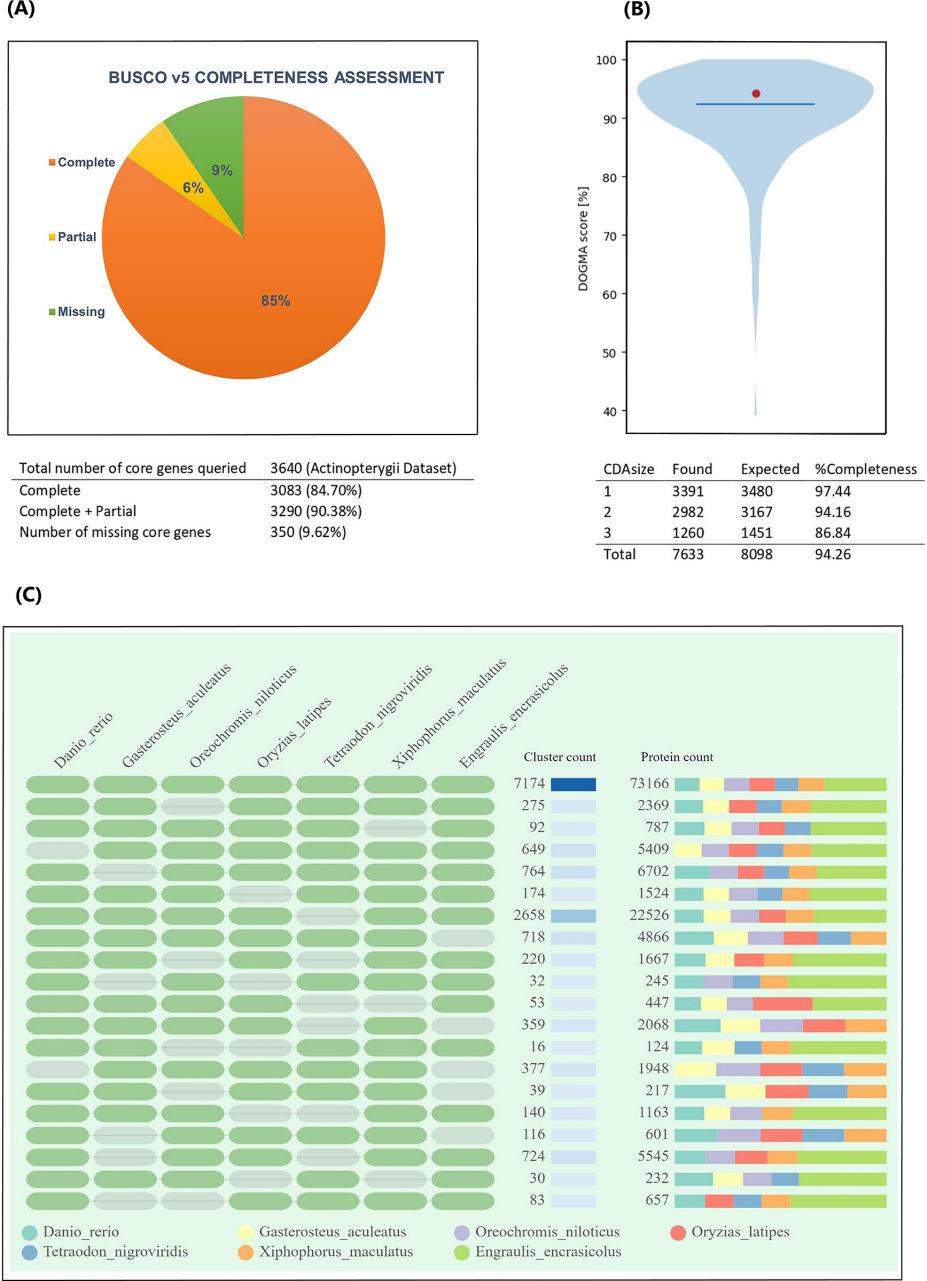
**(A)** The completeness assessment of *E*. *encrasicolus* assembled transcriptome using BUSCO v5 (fish specific Actinopterygii dataset), **(B)** The completeness of *de novo* assembled transcriptome using DOGMA Conserved Domain Arrangements (CDA) or "core set" from a vertebrate reference set, **(C)** The total number of protein clusters and counts found in *E*. *enrasicolus* and well-annotated fish proteomes.

The RNA-Seq approach was used to comprehensively characterize the *E*. *encrasicolus* transcriptome using five juveniles and various mature tissues (including liver, muscle, kidney, ovary, testis, gill, dorsal fin and cauda). After quality filtration, more than one billion clean paired-end reads 100 base pairs long yielded a total of 102.3 billion bases generated from anchovy tissue samples (S1 Table). The assembled transcriptome contained a total of 357,467 transcripts and 487,996,906 assembled bases with a GC percent of 53.14. The assembly’s N50 value was 1,536 bp, and the average transcript length was 1,365.15 bp (Table 1). A total of 285,327 predicted coding sequences were identified and BUSCO completeness assessment based on this dataset revealed 90.38% completeness (Fig 2A). DOGMA analysis, or domain-based transcriptome quality assessment, across 262 vertebrate proteomes indicates 94.26% completeness (Fig 2B). In another method we used to measure the completeness of assembled transcripts, we compared non-redundant peptides with well-annotated fish proteomes in the OrthoVenn [51] database and identified 7,174 orthologous clusters (E-value <10^−5^) between *E*. *encrasicolus* and well-annotated fish proteomes (Fig 2C).

**Table 1:** Summary statistics of *E*. *encrasicolus de novo* transcriptome assembly.

Sample	*E*. *encrasicolus*
Total Length (nt)	487,996,906
Sequence Number	357,467
Mean length (nt)	1,365.15
N50 sequence length (nt)	1,536
N90 sequence length (nt)	719
L50 sequence count	92,083
Longest sequence (nt)	49,711
Shortest sequence (nt)	531
The number of contigs >1Kb	181,035
The number of contigs >10Kb	332
GC%	47.11

The outcomes of these evaluations indicate that the *de novo* assembly is reliable for functional annotation and comparative transcriptomics. We also aligned non-redundant coding sequences and translated peptide sequences to well-known functional databases (Uniprot90/SwissProt, Gene Ontology (GO), KEGG Orthology (KO), PFAM) to obtain a comprehensive functional annotation of these transcripts. Of the 285,327 coding transcripts identified, 196,171 (68.75%), 172,418 (60.42%), 169,603 (59.4%), 95,464 (33.45%) and 72,560 (25.4%) were annotated against Uniprot90, PFAM, eggNOG 5.0, KO and GO databases, respectively. The taxonomic classification for each transcript based on DIAMOND similarity search showed *D*. *rerio* (29.73%), *O*. *niloticus* (9.94%), *X*. *maculatus* (5.41%), *G*. *aculeatus* (4.44%), *O*. *latipes* (3.39%), *T*. *nigroviridis* (2.74%), *T*. *rubripes* (2.73%) and *G*. *morhua* (2.46%).

### Detecting candidate genes under positive selection

**Fig 3:**
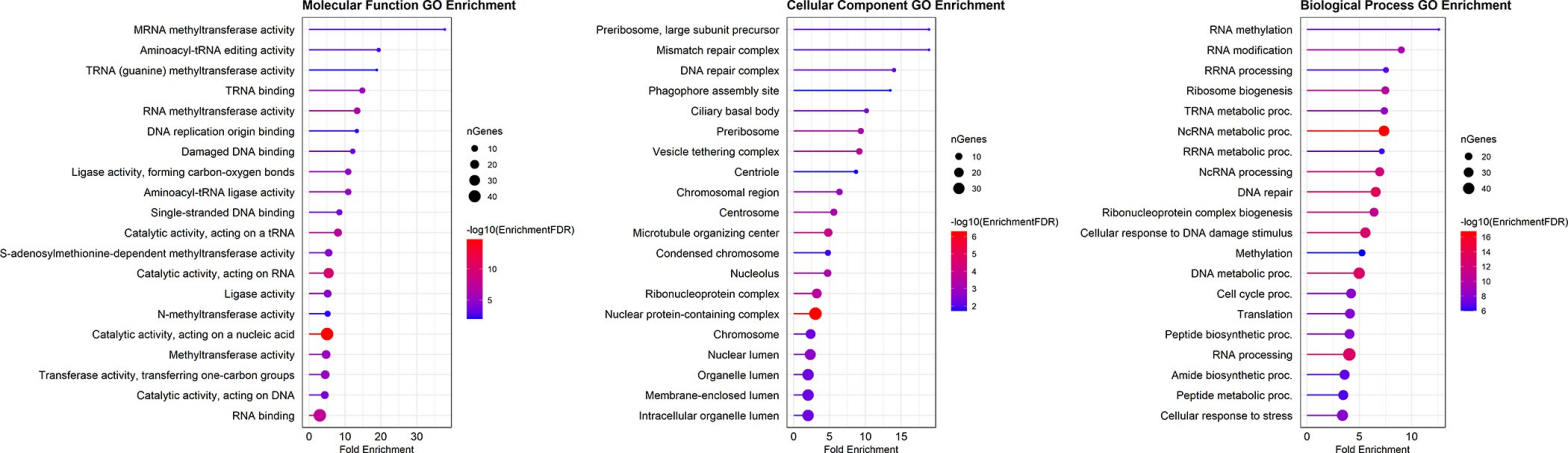
Functional GO enrichment of 535 positively selected genes in the *E*. *encrasicolus* transcriptome. The GO enrichment of PSGs was classified into three main GO categories: "Molecular function," "Biological process" and "Cellular component".

Following protein sequence alignment and gene family construction, we found 2,409 1:1 single-copy gene families for six fish (*D*. *rerio*, *G*. *aculeatus*, *O*. *niloticus*, *T*. *rubripes*, *T*. *nigroviridis*, *X*. *maculatus*). Orthologous sequences of *E*. *encrasicolus* were identified based on zebrafish single-copy genes using Reciprocal Best Hits (RBH) approaches and a total of 2,272 1:1 orthologs were ultimately obtained in seven fishes (S2 Table). Using the branch-site model, we identified a set of 535 genes containing positively selected sites, specifically in *E*. *encrasicolus* after multiple sequence alignment (S3 Table, S1 File). To evaluate the potential functions of these positively selected genes (PSGs), functional enrichment analysis was performed by mapping each PSG into GO database records with an adjusted *p*-value < 0.05 as significant enrichment. The results revealed that PSGs were substantially enriched in 255 unique GO terms. (S3 Table). Among biological process categories, we found that most PSGs were related to RNA methylation and modification, ribosome biogenesis, ncRNA processing, DNA repair, ribonucleoprotein complex biogenesis, DNA metabolic and cell cycle processing, and peptide/amide biosynthesis. In molecular function, the most highly represented functional clusters were mRNA methyltransferase, aminoacyl-tRNA editing, tRNA methyltransferase/binding, DNA replication and binding, ligase activity, and RNA binding. Under the cellular component, preribosome, mismatch, DNA repair complex, ciliary basal bodys and vesicle tethering complex were the most enriched subcategories, followed by chromosome, nucleus and organelle lumens (Fig 3A–3C).

**Fig 4:**
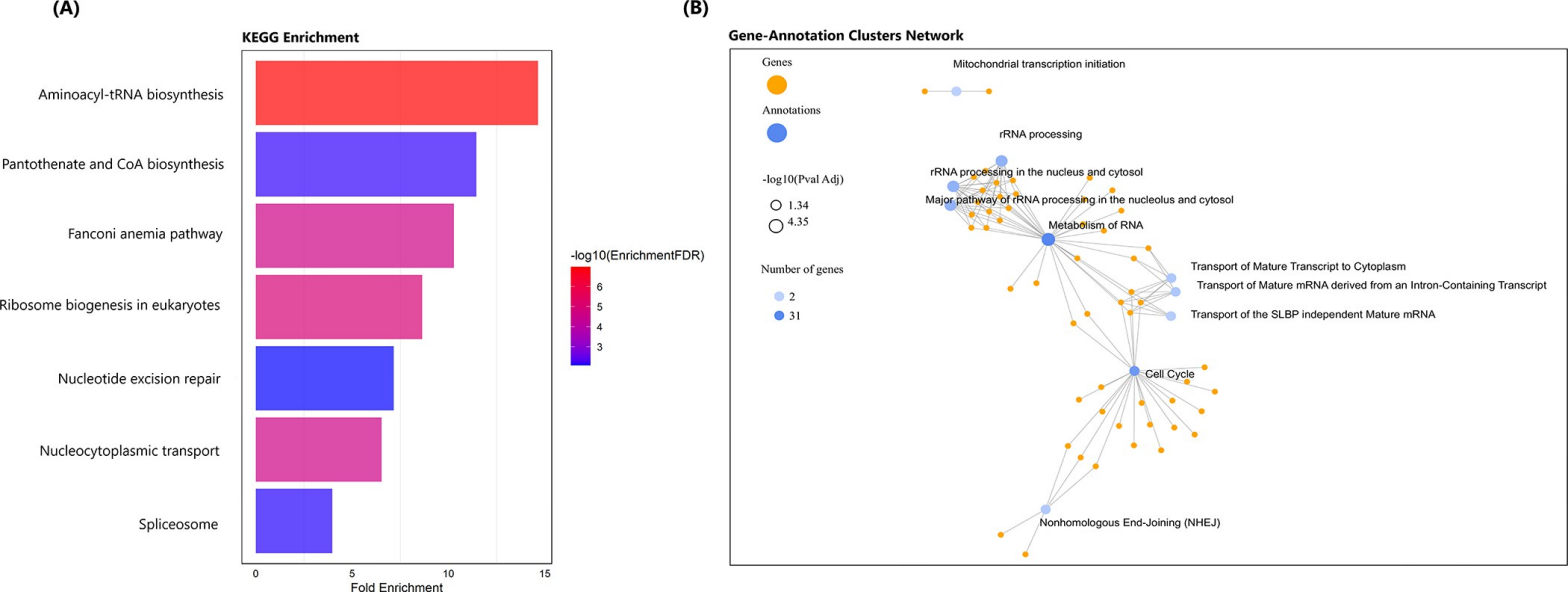
**(A)** Pathway assignment of 535 PSGs on the Kyoto Encyclopedia of Genes and Genomes (KEGG), **(B)** Funtional gene-annotation cluster network of PSGs.

KEGG pathway analysis revealed that five KEGG pathways were enriched in PSGs containing aminoacyl-tRNA biosynthesis, pantothenate and CoA biosynthesis, Fanconi anemia, ribosome biogenesis and nucleocytoplasmic transport (Fig 4A). RNA metabolism, rRNA processing and cell cycle annotations were enriched in gene-cluster analysis (Fig 4B).

**Fig 5:**
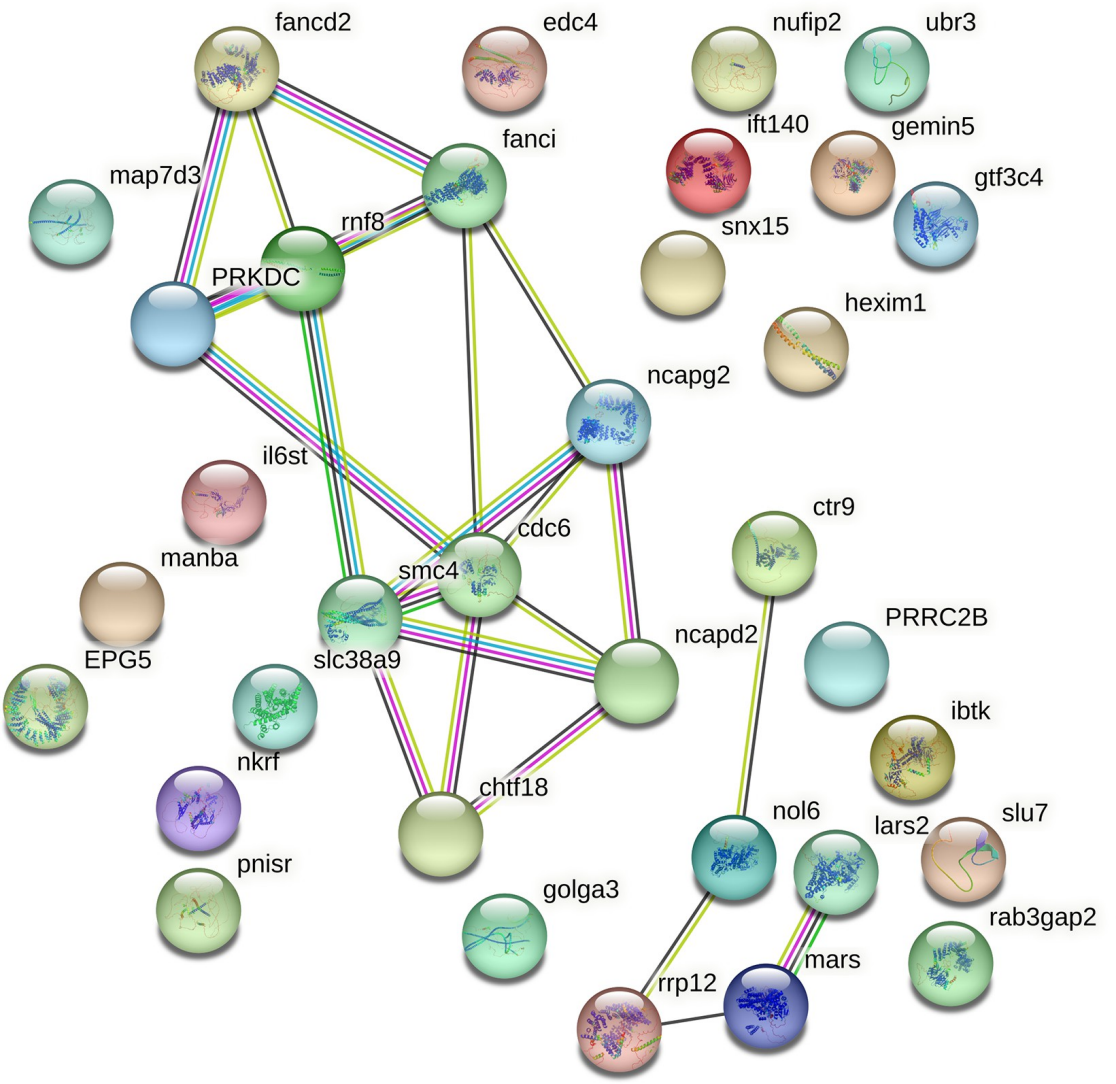
The PIP interactions of top statistically significant PSGs (turquoise and purple lines: Known interactions; green, red and blue lines: Predicted interactions; yellow line: Text mining; black line: Co-expression).

Furthermore, we analyzed the major functions of the top 35 genes containing statistically significant positively selected sites based on the NCBI Gene database. According to the results, these genes appeared to be primarily involved in various molecular and cellular processes, such as (i) RNA binding activity, mRNA processing, embryonic development, cell survival and differentiation, ribosome biogenesis/processing (*pnisr*, *prrc2b*, *nufip2*, *ibtk*, *nol6*, *rrp12*), (ii) DNA replication and repair (*cdc6*, *fanci*, *fancd2*), (iii) histone binding activity, chromosome condensation, cohesion and assembly (*ncapd2*, *chtf18*, *smc4*, *ncapg2*), (iv) pre-mRNA splicing, transcriptional repression and regulation (*slu7*, *gemin5*, *edc4*, *nkrf*, *ctr9*), and (v) signal transducing, intracellular trafficking, transmembrane transporter activity, transport of proteins and lipids (*il6st*, *snx15*, *golga3*, *slc38a9*) (Table 2). Furthermore, we carried out a PPI network analysis, and two main interaction patterns were identified in statistically significant PSGs (Table 2). First, strong PIP interactions were found among FANCD2, FANCI, PRKDC and RNF8, and the other was between CDC6, SMC4, NCAPG2, NCAPD2 and CHTF18 proteins (Fig 5).

**Table 2:** The description and function of the top thirty-five genes with statistically significant positively selected sites in *E*. *encrasicolus* (False Discovery Rate or FDR <0.0005).

Transcript ID	Zebrafish Ortho.	*P*-value	FDR	Positive_Selection_Sites	Function
*anchovy*.*40035*	*pnisr*	0	0	125 N 0.958*;212 A 0.997**;555 F 0.994**	PNN-interacting serine/arginine-rich protein
*anchovy*.*42934*	*prrc2b*	0	0	1980 T 0.998**;2546 Q 0.995**	Proline-rich coiled-coil 2B
*anchovy*.*10428*	*cdc6*	6.42E-06	0.000102602	749 Q 0.981*;555 G 0.993**	Cell division cycle 6 homolog
*anchovy*.*5864*	*ibtk*	7.99E-06	0.000125023	288 V 0.992**	Inhibitor Of Bruton Tyrosine Kinase
*anchovy*.*7276*	*si*:*ch211-259g3*.*4*	8.33E-06	0.000127686	1700 V 0.982*;688 E 0.984*	Bridge-like lipid transfer protein 2
*anchovy*.*25754*	*nkrf*	8.28E-06	0.000127718	551 I 0.995**;613 S 0.963*	NFKB repressing factor
*anchovy*.*41523*	*gtf3c4*	8.59E-06	0.000130693	84 V 0.970*;301 P 0.990*	General transcription factor IIIC
*anchovy*.*35961*	*fanci*	9.51E-06	0.000142673	571 A 0.972*;1331 S 0.991**	FA complementation group I
*anchovy*.*18750*	*ncapd2*	1.02E-05	0.0001508	981 S 0.992**	Non-SMC condensin I complex, subunit D2
*anchovy*.*6611*	*lars2*	1.07E-05	0.000156438	329 E 0.956*;226 T 0.982*	Leucyl-tRNA synthetase 2, mitochondrial
*anchovy*.*6288*	*il6st*	1.07E-05	0.000156627	770 T 0.962*;912 P 0.978*;873 Q 0.968*;809 T 0.981*;897 P 0.982*;335 A 0.964*	Interleukin 6 signal transducer
*anchovy*.*42246*	*ncapg2*	1.23E-05	0.000174171	857 E 0.968*	Non-SMC Condensin II Complex Subunit G2
*anchovy*.*27574*	*snx15*	1.22E-05	0.000174532	32 L 0.984*	Sorting nexin 15
*anchovy*.*30590*	*manba*	1.22E-05	0.000175523	291 V 0.971*	Mannosidase, beta A, lysosomal
*anchovy*.*42545*	*nol6*	1.26E-05	0.000177222	733 Q 0.987*;1124 G 0.983*;908 T 0.952*;338 S 0.978*	Nucleolar protein 6 (RNA-associated)
*anchovy*.*16061*	*slu7*	1.32E-05	0.000184617	632 P 0.989*;350 G 0.955*	SLU7 homolog, Splicing factor
*anchovy*.*40525*	*chtf18*	1.34E-05	0.000185272	962 S 0.988*;856 Q 0.972*	Chromosome transmission fidelity factor 18
*anchovy*.*40376*	*gemin5*	1.37E-05	0.000189329	430 R 0.960*;724 L 0.990**;357 S 0.976*	Gem associated protein 5
*anchovy*.*30480*	*rrp12*	1.42E-05	0.000194075	676 S 0.953*;922 M 0.981*	Ribosomal RNA processing 12
*anchovy*.*8919*	*mars*	1.44E-05	0.000196759	808 V 0.988*;569 A 0.971*	Methionyl-tRNA synthetase 1
*anchovy*.*42822*	*fancd2*	1.46E-05	0.000197527	1058 A 0.952*;1219 C 0.987*	FA complementation group D2
*anchovy*.*28076*	*epg5*	1.66E-05	0.000221575	1561 A 0.975*;2640 T 0.958*	Ectopic P-granules autophagy protein 5
*anchovy*.*2705*	*nufip2*	1.70E-05	0.00022536	240 R 0.957*;215 A 0.953*	Nuclear FMR1 interacting protein 2
*anchovy*.*17168*	*rnf8*	1.75E-05	0.000231664	135 E 0.968*	Ring finger protein 8
*anchovy*.*57632*	*ift140*	1.78E-05	0.000233078	837 S 0.968*;117 A 0.963*	Intraflagellar transport 140
*anchovy*.*14694*	*ubr3*	1.86E-05	0.000241171	1746 A 0.987*	Ubiquitin protein ligase E3 component
*anchovy*.*2822*	*edc4*	2.07E-05	0.000263823	1654 S 0.988*;1033 A 0.960*	Enhancer of mRNA decapping 4
*anchovy*.*16201*	*golga3*	2.06E-05	0.000264788	1281 T 0.981*	Golgin A3
*anchovy*.*56601*	*prkdc*	2.12E-05	0.00026738	4609 K 0.966*;3901 W 0.952*	Protein kinase, DNA-activated
*anchovy*.*42287*	*slc38a9*	2.16E-05	0.000270412	538 T 0.966*;347 K 0.995**	Solute carrier family 38 member 9
*anchovy*.*17574*	*ctr9*	2.20E-05	0.000273904	1110 Q 0.969*;1159 S 0.961*;801 S 0.982*	Paf1/RNA Poly II complex component
*anchovy*.*39808*	*hexim1*	2.31E-05	0.00028512	210 N 0.991**	HEXIM P-TEFb complex subunit 1
*anchovy*.*34555*	*map7d3*	2.44E-05	0.000299164	151 A 0.991**	MAP7 domain containing 3
*anchovy*.*23993*	*rab3gap2*	2.54E-05	0.00030956	1389 S 0.994**	RAB3 GTPase activating protein subunit 2
*anchovy*.*14788*	*smc4*	2.70E-05	0.000326043	1289 C 0.991**;86 T 0.986*;461 A 0.971*	Structural maintenance of chromosomes 4

### Gene expression pattern of positively selected genes

**Fig 6:**
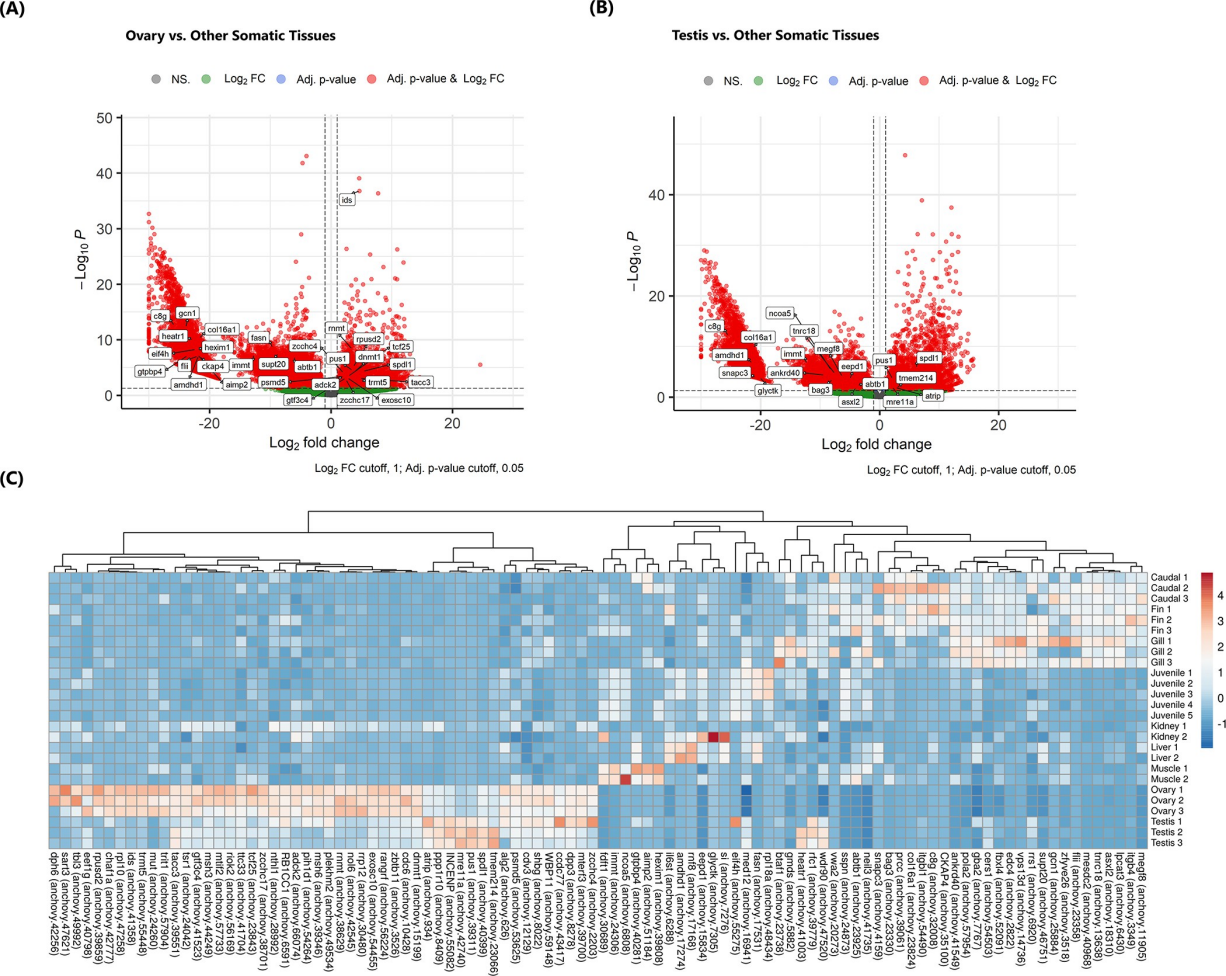
Volcano plot showing the differentially expressed genes in the ovary **(A)**, testis **(B)** and some positively selected genes shown in the box. **(C)** Heatmap showing the differential expression pattern of 100 positively selected genes in a tissue-specific manner.

Determining the gene expression levels in the reproductive tissues (ovary and testis) of the highly fecund *E*. *encrasicolus*, is valuable in detecting the genes related to reproduction. In this context, we identified genes whose expression levels were significantly different in the ovary and testis compared to other somatic tissues. Overall, 10,691 DEGs were identified, of which 1,039 were upregulated and 9,653 were downregulated in the ovary versus other somatic tissues(Fig 6A). A total of 11,395 DEGs between the testis and other somatic tissues were identified, consisting of 1,075 upregulated and 10,320 downregulated genes (Fig 6B).

Although many DEGs are present in both the ovaries and testicles, the majority are either non-coding or truncated transcripts. Because of this we focused on protein-coding DEGs in our research (S4 and S5 Tables). As for PSGs, a total of 58 genes were upregulated and 204 were downregulated in the ovary, while 70 were upregulated and 186 were downregulated in the testis of *E*. *encrasicolus* (S6 and S7 Tables). Because our study targets the ovarian transcriptome profile and dynamics of *E*. *encrasicolus*, we focused on genes that were both positively selected and differentially expressed in the ovary compared to somatic tissues and testis. A heatmap was drawn based on statistical significance and functional relevance, to explore the positively selected DEGs in a tissue-specific manner and investigate how the expression levels of positively selected genes changes in the ovary compared to other tissues. A list of top up-/downregulated and positively selected transcripts in ovarian tissue is provided in Table 3. In terms of their molecular functions in the cell, most upregulated PSGs involve RNA and DNA transferase, developmental transcription factors, protein kinases and replication factors, whereas downregulated genes seem to be involved in the synthesis functions of structural proteins such as collagen, fatty acid biosynthesis and sex hormone-binding globulin (Table 3). In teleost reproduction, iduronate-2-sulfatase (*ids*) and nucleotide methyltransferases (*dnmt1* and *rnmt*) are notable among the differentially expressed positive selection genes (PSGs).

**Table 3:** Top most up- and downregulated positive selected genes in ovary of *E*. *encrasicolus* (adjusted P value; Padj).

Transcript ID	Zebrafish Ortho.	Log_2_FC	Padj	Regulation	Annotation
*anchovy*.*41358*	*ids*	4,64	1,63E-37	Up ▲	Iduronate 2-sulfatase, GO:00044239; Sulfatase activity
*anchovy*.*38629*	*rnmt*	3,35	3,83E-09	Up ▲	RNA Guanine-7 Methyltransferase, GO:0008168; Methyltransferase activity
*anchovy*.*5758*	*zgc*	2,29	1,09E-07	Up ▲	**-**
*anchovy*.*55082*	*incenp*	4,42	1,31E-06	Up ▲	Inner centromere protein, GO:0000070; Mitotic sister chromatid segregation
*anchovy*.*39859*	*rpusd2*	3,71	5,92E-06	Up ▲	RNA pseudouridine synthase domain containing 2, GO:0003723; RNA binding
*anchovy*.*39311*	*pus1*	3,47	2,88E-05	Up ▲	Pseudouridine synthase 1, GO:0009982; Pseudouridine synthase activity
*anchovy*.*40399*	*spdl1*	5,32	4,53E-05	Up ▲	Spindle apparatus coiled-coil protein 1, GO:0051301; Cell division
*anchovy*.*15199*	*dnmt1*	3,81	6,79E-05	Up ▲	DNA (cytosine-5-)-methyltransferase 1, GO:0003682; chromatin binding
*anchovy*.*41735*	*neil3*	3,86	6,88E-05	Up ▲	Nei-like DNA glycosylase 3, GO:0003677; DNA binding
*anchovy*.*2203*	*zcchc4*	1,94	7,63E-05	Up ▲	Zinc finger, CCHC domain containing 4, GO:0003676; nucleic acid binding
*anchovy*.*39779*	*rfc1*	2,69	8,65E-05	Up ▲	Replication factor C (activator 1) 1, GO:0003677; DNA binding
*anchovy*.*23151*	*si*	1,82	0,000226333	Up ▲	**-**
*anchovy*.*23843*	*tcf25*	2,92	0,000302828	Up ▲	Transcription factor 25 (basic helix-loop-helix),
*anchovy*.*53625*	*psmd5*	2,02	0,000402343	Up ▲	Proteasome 26S subunit, GO:0043248; proteasome assembly
*anchovy*.*66074*	*adck2*	1,49	0,000712454	Up ▲	AarF domain containing kinase 2, GO:0004672; protein kinase activity
*anchovy*.*54455*	*exosc10*	2,79	0,000844875	Up ▲	Exosome component 10, GO:0003676; nucleic acid binding
*anchovy*.*39551*	*tacc3*	4,83	0,000883696	Up ▲	Transforming, acidic coiled-coil containing protein 3, GO:0007052; mitotic spindle organization
*anchovy*.*41523*	*gtf3c4*	2,27	0,000936517	Up ▲	General transcription factor IIIC
*anchovy*.*55448*	*trmt5*	5,03	0,001279193	Up ▲	tRNA methyltransferase 5, GO:0008168; methyltransferase activity
*anchovy*.*38701*	*zcchc17*	2,50	0,001913984	Up ▲	Zinc finger, CCHC domain containing 17, GO:0003723; RNA binding
*anchovy*.*32008*	*c8g*	-25,91	2,23E-13	Down ▼	Complement component 8, GO:0036094; small molecule binding
*anchovy*.*8022*	*shbg*	-24,21	2,84E-13	Down ▼	Sex hormone-binding globulin, GO:0005497; androgen binding
*anchovy*.*25884*	*gcn1*	-23,88	4,11E-13	Down ▼	GCN1 activator of EIF2AK4; GO:0019901; protein kinase binding
*anchovy*.*56169*	*riok2*	-23,67	1,16E-12	Down ▼	RIO kinase 2, GO:0005524; ATP binding
*anchovy*.*8409*	*ppp1r10*	-23,51	5,43E-12	Down ▼	Protein phosphatase 1, regulatory subunit 10, GO:0046872; metal ion binding
*anchovy*.*23824*	*col16a1*	-21,61	4,48E-11	Down ▼	Collagen, type XVI, alpha 1, GO:0005201; extracellular matrix structural constituent
*anchovy*.*41003*	*heatr1*	-22,87	1,14E-10	Down ▼	HEAT repeat containing 1, GO:0043066; negative regulation of apoptotic process
*anchovy*.*17531*	*fasn*	-9,70	2,15E-09	Down ▼	Fatty acid synthase, GO:0006633; fatty acid biosynthetic process
*anchovy*.*25602*	*osmr*	-5,24	5,34E-09	Down ▼	Oncostatin M receptor, GO:0004896; cytokine receptor activity
*anchovy*.*55275*	*eif4h*	-22,3	5,43E-09	Down ▼	Eukaryotic translation initiation factor 4h, GO:0097010; eukaryotic translation initiation factor 4F complex assembly

The differential expression profile of PSGs in the testis falls outside of the scope of this study; however, we noticed that some genes were commonly upregulated or downregulated in both ovary and testis. For example, some differentially expressed PSGs, such as *cntln* (centrosomal protein), *pola2* (DNA directed polymerase), *rfc1* (replication factor C), *chaf1a* (chromatin assembly factor 1) and *spdl1* (spindle apparatus coiled-coil protein 1) involved in biological processes such as DNA polymerase, cell division and chromosomal assembly, were upregulated in both ovaries and testicles. In contrast, genes such as *mrpl39* (mitochondrial ribosomal protein), *fga* (fibrinogen alpha chain), *gtpbp4* (GTP binding protein), *fggy* (FGGY carbohydrate kinase), *vps16* (vacuolar protein sorting 16), *mtmr14* (myotubularin related protein 14), *lepr* (leptin), *itgb4* (integrin 4) and *ckap5* (cytoskeleton-associated protein 5), mainly responsible for the synthesis of mostly structural proteins, were found to be downregulated in both the ovary and testis (S6 and S7 Tables).

### The expression profile of germ cell development and oocyte maturation genes in ovary tissue

**Fig 7:**
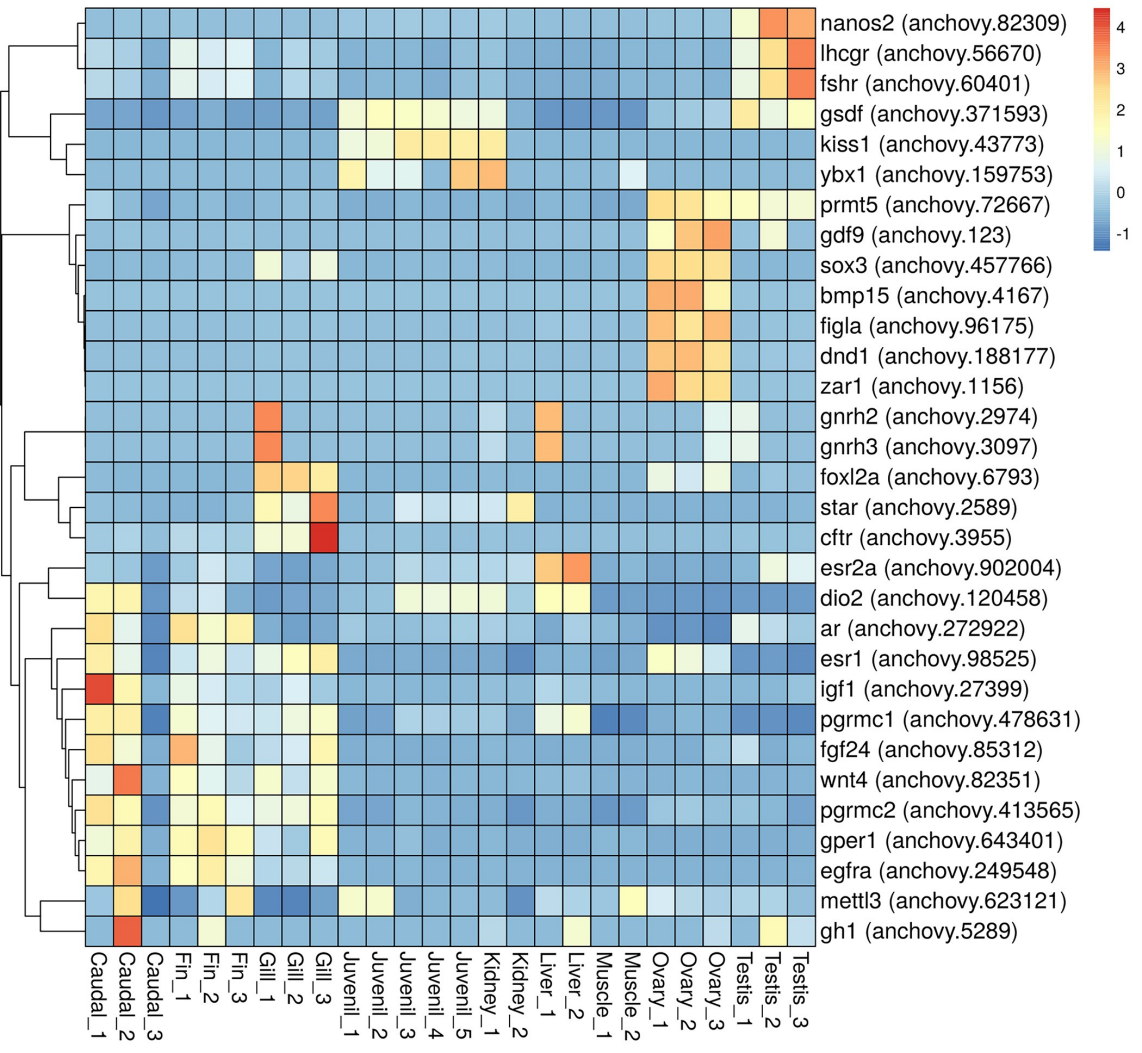
The heatmap showed that the expression level of thirty-one genes is involved in ovary differentiation, growth, maturation, and maintenance across *E*. *encrasicolus* tissues.

In addition to assessing the expression of genes under positive selection in ovarian tissue, the expression profile of genes involved in reproduction and egg development was investigated in *E*. *encrasicolus* tissues. These genes involved in ovarian development were split into five categories: (i) *igf1* (insulin-like growth factor 1, anchovy.27399), *egfra* (epidermal growth factor receptor a, anchovy.249548), *nanos2* (nanos homolog 2, anchovy.82309), *prmt5* (protein arginine methyltransferase 5, anchovy.72667), *cftr* (CF transmembrane conductance regulator, anchovy.3955), *dnd1* (DND microRNA-mediated repression inhibitor 1, anchovy.188177), and *fgf24* (fibroblast growth factor 24, anchovy.85312) in primordial and somatic germ cell development, (ii) *figla* (folliculogenesis specific bHLH transcription factor, anchovy.96175) and *wnt4* (wingless-type MMTV integration site family, member 4, anchovy.82351) in from cyst to individual oocyte transition, (iii) *fshr* (follicle stimulating hormone receptor, anchovy.60401), *gh1* (growth hormone 1, anchovy.5289), *dio2* (iodothyronine deiodinase 2, anchovy.120458), *foxl2a* (forkhead box L2a, anchovy.6793), and *zar1* (zygote arrest 1, anchovy.1156) in follicle activation, (iv) *esr1* (estrogen receptor 1, anchovy.98525), *esr2a* (estrogen receptor 2a, anchovy.902004), *gper1* (G protein-coupled estrogen receptor 1, anchovy.643401), *gdf9* (growth differentiation factor 9, anchovy.123), *bmp15* (bone morphogenetic protein 15, anchovy.4167), *ar* (androgen receptor, anchovy.272922), *gsdf* (gonadal somatic cell derived factor, anchovy.371593), and *sox3* (SRY-box transcription factor 3, anchovy.457766) in follicle growth and vitellogenesis, and (v) *lhcgr* (luteinizing hormone/choriogonadotropin receptor, anchovy.56670), *kiss1* (KiSS-1 metastasis suppressor, anchovy.43773), *gnrh2* (gonadotropin-releasing hormone 2, anchovy.2974), *gnrh3* (gonadotropin-releasing hormone 3, anchovy.3097), *star* (steroidogenic acute regulatory protein, anchovy.2589), *pgrmc1* (progesterone receptor membrane component 1, anchovy.478631), *pgrmc2* (progesterone receptor membrane component 2, anchovy.413565), *mettl3* (methyltransferase like 3, anchovy.623121), and *ybx1* (Y box binding protein 1, anchovy.159753) in follicle oocyte maturation. The normalized heatmap showed that there were differences in the expression profile of seven genes (*prmt5* (anchovy.72667), *gdf9* (anchovy.123), *sox3* (anchovy.457766), *bmp15* (anchovy.4167), *figla* (anchovy.96175), *dnd1* (anchovy.188177), and *zar1* (anchovy.1156)) in ovary compared to other tissues (Fig 7). Furthermore, the genes nanos2 (anchovy.82309), lhcgr (anchovy.56670), and fshr (anchovy.60401) exhibit higher expression levels in testicular tissues compared to other tissues (Fig 7).

## Discussion

Revealing molecular processes in gonad development of teleosts is a crucial part of numerous investigations of fish reproduction and development [52]. Detecting complex multi-factor regulatory factors, particularly during ovarian development and differentiation, by transcriptomic methods is essential for elucidating fish reproductive biology. The ovary is highly conserved transcriptionally throughout animal taxa, from invertebrates to vertebrates. Studies have shown that fish oogenesis, or the generation of eggs, and oocyte maturation are complex processes regulated by a wide range of intra- and extra-ovarian factors [53]. Intra-ovarian factors, specifically transcriptomic dynamics, have been explored through recent genomic and small RNA profiling studies, which have contributed significantly to our understanding of the regulation of oocyte growth and development, ovary maturation, sex determination and egg quality [54–57]. In this context, the genome-wide transcriptome analyses of gonadal and somatic tissues of fishes characterized by high fecundity, like anchovy, may facilitate the identification of genes related to egg production, multiple-spawning and ovary development.

Compared to fish species with well-annotated genomes and generally low fecundity, *pnisr* (*anchovy*.*40035*), *prrc2b* (*anchovy*.*42934*) and *cdc6* (*anchovy*.*10428*) had the most statistically significant positive selection signatures in *E*. *encrasciolus*. A member of a multi-protein complex in the nucleus, *pnisr* (PNN-interacting serine/arginine-rich protein) is involved in the processing of pre-mRNA and has been linked to the regulation of cell proliferation and differentiation [58]. The proline-rich coiled-coil 2B (*prrc2b*) is a highly conserved gene found in many vertebrate genomes [59]. The *prrc2b* is an mRNA-binding protein identified in multiple cell types and its putative arginine-glycine (RG)-rich domains have been documented to interact with RNA. Furthermore, it has been suggested that *prrc2b* is part of the eukaryotic initiation factor 4G2 (eIF4G2)-mediated translation initiation complex [60,61]. A recent study showed that the translation of specific proteins called oncogenes and cell cycle regulators, like ccnd2, decreases when *prrc2b* is knocked-down, as revealed by polysome-associated RNA-seq. The decrease in these proteins leads to a reduction in the process of G1/S phase transition, which is necessary for cell division and growth. Finally, researchers concluded that *prrc2b*, an RNA binding protein, plays a key role in translating specific proteins needed for cell cycle progression and cell proliferation [62]. As another essential protein involved in the division of proliferative cells, the cell division cycle 6 (Cdc6) protein is critical for initiating DNA replication by facilitating the assembly of pre-replicative complexes (pre-RCs) at replicative origins during the G1 phase of the cell cycle [63]. Research has shown that the expression of cdc6 is a critical factor in the ability of primary oocytes to replicate DNA. It plays an essential role in cell cycle regulation during oogenesis. Mainly, Cdc6 helps to inhibit the S-phase of the cell cycle between the first and second meiotic M-phases, and to control the entry and exit of cells into and out of the M-phase through the inhibition of CDK1 [64,65]. Another study investigated the role of Cdc6 in JH (juvenile hormone)-dependent vitellogenesis and oogenesis because Cdc6 is required for the formation of the prereplication complex. As a result, the expression of vitellogenin in the fat body is significantly decreased due to Cdc6 knockdown, and oocyte maturation and ovarian development are hampered considerably [66]. Another seminal work showed that Cdc6 is required to recruit the minichromosome maintenance (MCM) helicase to the pre-replication complex. In *Xenopus* oocytes, the production of Cdc6 protein during maturation can be disrupted by inhibiting translation or injecting oligonucleotide antisense. Experimental evidence using recombinant Cdc6 protein demonstrates that translation of Cdc6 is both required and sufficient for allowing the egg to replicate its DNA before fertilization [67]. In line with previous studies, our findings suggest that these three PSGs (*pnisr*, *prrc2b*, and *cdc6*) probably play a role in the high reproduction capacity of highly fecundate teleost fishes.

The *fancd2* (FA complementation group D2) and *fanci* (Fanconi anemia, complementation group I) were identified as notable PSGs in our protein–protein interaction analysis, showing strong relationships with *prkdc* and *rnf8* genes. The cellular functions of these genes are mostly related to the repair of spontaneous DNA damage and DNA crosslink. A study on the function of FANCD2 in DNA damage responses during specific developmental stages in *Caenorhabditis elegans* demonstrated that a mutant strain with a deletion in the gene encoding the FANCD2 homolog, FCD-2, exhibited impaired egg laying, premature oogenesis and partial fertilization defects. These findings suggest that the FANCD2 homolog in *C*. *elegans* plays an important role in repairing spontaneous DNA damage and DNA crosslinks in proliferating and pachytene stage cells, and may also have a role in repairing double-stranded DNA breaks during embryogenesis [68]. On the other hand, Nie et al. [69] explored the interaction between FANCD2, a vital component of the Fanconi Anemia pathway, and several critical components of the Prmt5/piRNA pathways that are exclusive to germ cells and regulate the suppression of transposable elements (TEs). Using Pou5f1-eGFP reporter mice, which identify pure populations of primordial germ cells (PGCs), the authors found that a deficiency in FANCD2 leads to the unrepression of TEs, a depletion of PGCs, and defects in spermatogenesis and oogenesis. This suggested that the Fanconi Anemia pathway plays a role in TE repression in early PGCs, likely through a mechanism involving the promotion of repressive H2A/H4R3me2s marks on TEs by FANCD2 and the acceleration of this process by Prmt5 [69]. An important finding is that FANCI, a protein involved in Fanconi anemia, interacts with proteins that function in ribosome biogenesis, the process of synthesizing ribosomes in cells, in addition to DNA repair [70]. In another critical study conducted, knockout of five FA genes (*fanca*, *fancb*, *fancm*, *fanco* and *fancq*) did not affect the oogenesis process in zebrafish; however, when 12 other genes, including *fanci*, were knocked out, almost no females remained among the surviving homozygous knockouts [71]. The identification of DNA repair and ribosome biogenesis genes in highly fecund fish species, such as anchovy, can contribute to a greater understanding of teleost oogenesis and reproduction through the examination of the necessary mechanisms for high fecundity.

Among PSGs in *E*. *encrasicolus* that differ significantly in gene expression, iduronate-2-sulfatase (*ids*) was determined to be a prominent gene. Biochemical and loss of function experiments utilizing the antisense morpholino technology were conducted to investigate the potential role of *ids* in zebrafish embryonic development. The results of this study demonstrated that *ids* is highly expressed in the early stages of development and that its functional knockdown significantly disrupts early development, resulting in abnormal anterior-posterior patterning and various morphological defects [72]. In another study, Bellesso et al. utilized CRISPR/Cas9 technology to create a transgenic zebrafish line with a five-base-pair deletion in the *z-ids* gene, leading to a premature stop codon at amino acid 118 and a truncated form of z-IDS in cells. This loss of z-IDS function during early development decreased Fgf signaling and negatively impacted bone development at later stages [73]. Other notable PSGs exhibiting differentially expressed patterns in ovary tissue are methyltransferases (*rnmt*, *dnmt1*, *trmt5*) and it is well-known that oocyte growth is accompanied by dynamic epigenetic modifications [74]. Among methylases, DNA methylases are known to be essential for development and reproduction. Methylation of cytosine bases in DNA is initiated by the *de novo* methylases Dnmt3a and Dnmt3b, while the maintenance of this methylation after DNA replication and repair is dependent on the action of the maintenance methylase Dnmt1 specifically recognizing hemimethylated DNA duplexes and transferring the methylation pattern of the parental strand onto the newly synthesized strand. Although mice lacking the Dnmt1 enzyme do not survive past 9.5 days of embryonic development, zebrafish homozygous for a mutant form of the *dnmt1* gene encoding an enzyme with impaired catalytic activity do not survive beyond eight days post fertilization. The *dnmt1* mRNA is found in large quantities in the egg and is responsible for maintaining methylation after each cell division [75–78]. Dnmt1 is required for pre-gastrula zebrafish development [79] and *dnmt1* was reportedly highly expressed in the gonads of mandarin fish (*Siniperca chuatsi*) [80]. These observations demonstrate the essential role of Dnmt1 in zebrafish and teleost development. In addition to gonadal development, studies have shown that *dnmt1* is involved in developing somatic tissues in zebrafish. Dnmt1 is necessary for developing the zebrafish auditory organ through regulation of cell cycle genes in conjunction with the Wnt and Fgf signaling pathways. Besides, Dnmt1 helps to sustain the proliferation, gene expression and integration into the retina of daughter cells derived from retinal stem cells [81,82]. According to our findings, another differentially expressed PSG is the *rmtf* (RNA guanine-7 methyltransferase) gene, classified among Cap methyltransferases. The cap homeostasis process involves removing and replacing caps and impacts a portion of the mRNA transcriptome. The basic structure of the cap is generated through the methylation of the transferred guanosine at the N7 position by RNMT, which is necessary for proper mRNA processing and function [83]. The expression of rmtf is related to transcriptional activity [84] and the upregulation of RNMT coordinates mRNA capping and enhances ribosome abundance and translation capacity [85]. Downregulation of the methylation GO term, including rnmt, was observed in zebrafish gonads in response to environmental perturbations [86]. All these findings show that nucleotide methyltransferases (DNA and RNA) play a vital role in teleost reproduction (particularly in early stages) and organ development.

In our findings, two genes (*edc4* and *ctr9*) associated with oogenesis were identified among PSGs, and these genes seem to be involved in RNA synthesis or decay. Edc4 is a core component of processing (P)-bodies and helps maintain their integrity by serving as a scaffold for the assembly of the decapping enzyme DCP2 and its coactivator DCP1a [87,88]. Edc4 has been found to interact with the RNA binding protein MARF1 (meiosis regulator and mRNA stability factor 1) and this endonuclease promotes oogenesis. Recent studies have shown that knockout or impaired RNase activity of the MARF1 (interacting with Edc4) gene in female mice leads to a halt in meiosis during oogenesis and significant changes to the transcriptome [89,90]. As for CTR9, it is a component of the RNA polymerase II-associated factor 1 complex (PAF1C) along with LEO1, RTF1, PAF1 and CDC73 proteins, which are involved in the regulation of transcription and chromatin structure by RNA polymerase II. A recent study on *C*. *elegans* found that all components of the PAF1C complex, including CTR9, are essential for promoting oogenesis and their expression in germ cells is necessary for the development of oogenesis [91]. The *ctr9* gene generally has diverse cell proliferation, development and tumorigenesis functions. An example of the effects of Ctr9 mutation can be seen in zebrafish, where it leads to faulty somite development [92]. In mice, Ctr9 plays a role in maintaining genomic imprinting during gametogenesis. In *Drosophila*, a blastocyst without Ctr9 could not form an outgrowth, and Oct4 expression required for differentiating inner cell mass to parietal endoderm cells was found to be dependent on Ctr9 [93]. Moreover, CTR9 was discovered to be a central regulator of estrogen and may act as a tumor suppressor [94].

Besides genes undergoing positive selection, there is growing evidence indicating the involvement of numerous genes in ovary differentiation, growth, maturation, maintenance, and vitellogenesis [95–97]. In the context of our study, we observed that the expression levels of seven coding genes, namely *prmt5* (anchovy.72667), *gdf9* (anchovy.123), *sox3* (anchovy.457766), *bmp15* (anchovy.4167), *figla* (anchovy.96175), *dnd1* (anchovy.188177), and *zar1* (anchovy.1156), increased significantly in the ovary tissue of *E*. *encarsicolus*. In investigations into the effects of gene deficiencies on ovarian development in *D*. *rerio*, it was found that the *prmt5* and *dnd1* genes are involved in the development of primordial and somatic germ cells. When *prmt5* is absent, the survival rate is low, and all individuals that develop into adults are infertile males [98]. Disruption of the *dnd1* gene results in the development of infertile males in adults in *D*. *rerio* and causes deficiency of primordial germ cells and infertility in mice [99,100]. The *bmp15* and *gdf9* genes are involved in follicle growth and vitellogenesis. Disruption of *bmp15* leads to arrest at stage II (meiosis arrested at prophase I), followed by sex-reversal to the testis. However, the disruption of *gdf9* does not cause any defects in fertility or ovarian development in *D*. *rerio* [101]. The disruption of the *figla* gene inhibits the transition from cystic stage IA oocytes to individual follicular oocytes in *D*. *rerio* [102]. However, in mice, research showed that the disruption of the *figla* gene leads to massive depletion of oocytes and the failure of primordial follicle formation [103]. The genes *sox3* and *zar1* play important roles in follicle oocyte maturation and follicle activation, respectively. Disruption of *sox3* results in reduced fertility and a lower number of follicles at the vitellogenic stage in *D*. *rerio* [104]. Similarly, in mice, *sox3* disruption leads to reduced fertility, excess follicular atresia, and ovulation of defective oocytes[105]. In *D*. *rerio*, the disruption of the *zar1* gene has been found to impede oocyte growth beyond stage II and lead to the development of aberrant cortical granules [106]. Similarly, in mice, the disruption of *zar1* results in infertility due to a delay in germinal vesicle breakdown and first polar body emission [107]. These findings suggest the important role of *zar1* in regulating oocyte growth and maturation in teleosts and, more generally, vertebrates. Given that the expression levels of these genes are high in the ovary of *E*. *encrasicolus*, it is plausible that they play a crucial role in regulating ovarian development and, therefore, may be linked to the high fecundity observed in *Engraulidae* fish. We suggest that these genes could serve as potential targets for further fish reproduction investigations into the molecular mechanisms underlying the reproductive success of this species. We want to highlight that despite our extensive homology-based sequence analysis at the CDS and proteome levels, we were unable to identify genes in anchovy related to primordial and somatic germ cell development (*igf3*) [108], follicle activation (*fsbh*) [109], and oocyte maturation (*lhb* and *kiss2*) [110,111], which have been reported in zebrafish. This could be attributed to the lack of a complete anchovy genome.

Overall, it appears that the positively selected genes in *E*. *encrasicolus* are primarily involved in embryonic development, oogenesis, cell division and differentiation, DNA repair, mRNA synthesis, ribosome biogenesis and abundance. Therefore, analyzing the tissue-specific transcript profiles in high-fecundity fish species and comparing transcriptomes may help to identify potential reproductive-related gene candidates in vertebrates. Additionally, a comprehensive transcriptome study, particularly of *E*. *encrasicolus*, may contribute to our understanding of the reproductive biology of Clupeid fish species. To gain insight into the egg production capacity in fish species known for their high fecundity such as anchovy from an evolutionary standpoint, we recommend to investigate gonadal and somatic tissues with an increased number of samples per group to facilitate more biological inferences. Such an approach will contribute to a more comprehensive understanding of the underlying mechanisms and evolutionary adaptations associated with egg production in these species.

## Conclusions

Studies on the ovarian transcriptome in vertebrates, particularly in teleosts, have increased in recent years; however, these studies have primarily focused on egg quality, sex determination and ovarian development. Within the scope of this study, the positively selected genes and their expression levels in *E*. *encrasicolus*, which is characterized by high fecundity, were investigated for the first time using tissue-specific transcriptome analysis. Taken as a whole, our results provide potential gene candidates that may be associated with the high egg production capacity and reproduction observed in teleosts. Additionally, the comprehensive transcriptome profile of *E*. *encrasicolus* presented in our study will be a valuable resource supporting further investigations into processes such as oogenesis, oocyte maturation and ovarian development in fishes.

## Supporting information

S1 Table Sequencing summary statistics of tissue-specific anchovy transcriptome. (XLSX)

S2 Table The 2,272 1:1 single-copy orthologs in seven teleost species. (XLSX)

S3 Table A total of 535 positively selected genes in *E*. *engrasicolus* and their functional annotations. (XLSX)

S4 Table The differential expression of protein-coding genes in ovary tissues versus other somatic tissues (cauda, gill, fin, muscle, liver, kidney) and testis. (XLSX)

S5 Table The differential expression of protein-coding genes in testis versus other somatic tissues (cauda, gill, fin, muscle, liver, kidney) and ovary. (XLSX)

S6 Table The list of positively selected genes with altered expression in *E*. *encrasicolus* ovary. (XLSX)

S7 Table The list of positively selected genes with altered expression in *E*. *encrasicolus* testis. (XLSX)

S1 File Multiple alignment files of each 535 single-copy orthologous genes. (ZIP)

S2 File All analysis softwares and scripts used in this study. (DOCX)

S3 File The assessment of cross-platform variation between Illumina HiSeq2000 and MGI DNBSEQ-400 platforms regarding base quality, read lengths, sequencing depth, and sample clustering. (DOCX)
