# Supplementary material for: Global expression pattern of genes containing positively selected sites in European anchovy (Engraulis encrasicolus L.) may shed light on teleost reproduction
Source: PLoS One. 2023 Aug 11;18(8):e0289940. doi: 10.1371/journal.pone.0289940 (PMC10420382; doi:10.1371/journal.pone.0289940)
Supplement: S2 File — (DOCX) [file pone.0289940.s009.docx]

This document serves as Supplementary File to **Global expression pattern of genes containing positively selected sites in European Anchovy (*Engraulis encrasicolus* L.) may shed light on teleost reproduction**. Here, we present the scripts utilized for our analysis through the command line interface. Analyzes were carried out at the National Center for High Performance Computing of Turkey (UHeM). Most of tools were installed through Conda using the BioConda, Github repository and Singularity containers are also used during installation.

***#QC_Analysis***

| pwd  /okyanus/users/veldem/01.Direct_Projects/07.Anchovy_Transcriptome  cd 07.Anchovy_Transcriptome  fastqc *.fastq.gz  multiqc .  seqkit stats -a -T *.fastq.gz -j 28  fastp -i [Sample_Name]_1.fastq.gz -I [Sample_Name]_2.fastq.gz -o [Sample_Name]_1.clean.fastq.gz -O [Sample_Name]_2.clean.fastq.gz -q 20 -l 50 -w 16 |
| --- |

***#De_novo_Transcriptome_Assembly_and_Annotation***

| pigz –d –p 28 *.fastq.gz  cat *_1.clean.fastq > anchovy_forward.fastq  cat *_2.clean.fastq > anchovy_reverse.fastq  singularity exec –e trinityrnaseq.v2.15.1.simg Trinity --seqType fq --left anchovy_forward.fastq --right anchovy_reverse.fastq --max_memory 400G --CPU 24 --min_contig_length 300 --output trinity_anchovy_out_dir  gVolante Online Usage [https://gvolante.riken.jp/analysis.html]  DOGMA Online Usage [https://domainworld-services.uni-muenster.de/dogma/] |
| --- |

***#Alignment_and_DEG_Analysis***

| perl trinityrnaseq-v2.15.1/util/align_and_estimate_abundance.pl --prep_reference --transcripts trinity_anchovy.Trinity.fasta --seqType fq --samples_file **sample_meta_data.txt** --est_method RSEM --aln_method **bowtie2** --gene_trans_map trinity_anchovy.Trinity.fasta.gene_trans_map  **#sample_meta_data**  Caudal Caudal_rep1 C1_1.fq.gz C1_2.fq.gz  Caudal Caudal_rep2 C2_1.fq.gz C2_2.fq.gz  Caudal Caudal_rep3 C3_1.fq.gz C3_2.fq.gz  Fin Fin_rep1 F1_1.fq.gz F1_2.fq.gz  Fin Fin_rep2 F2_1.fq.gz F2_2.fq.gz  Fin Fin_rep3 F3_1.fq.gz F3_2.fq.gz  Gill Gill_rep1 G1_1.fq.gz G1_2.fq.gz  Gill Gill_rep2 G2_1.fq.gz G2_2.fq.gz  Gill Gill_rep3 G3_1.fq.gz G3_2.fq.gz  Ovary Ovary_rep1 Ov1_1.fastq.gz Ov2_2.fastq.gz  Ovary Ovary_rep2 Ov2_1.fastq.gz Ov2_2.fastq.gz  Ovary Ovary_rep3 Ov3_1.fastq.gz Ov3_2.fastq.gz  Testis Testis_rep1 Te1_1.fastq.gz Te1_2.fastq.gz  Testis Testis_rep2 Te2_1.fastq.gz Te2_2.fastq.gz  Testis Testis_rep3 Te3_1.fastq.gz Te3_2.fastq.gz  Juvenile Juvenile_Rep1 Juv1_1.fastq.gz Juv1_2.fastq.gz  Juvenile Juvenile_Rep2 Juv1_2.fastq.gz Juv2_2.fastq.gz  Juvenile Juvenile_Rep3 Juv1_3.fastq.gz Juv3_2.fastq.gz  Juvenile Juvenile_Rep4 Juv1_4.fastq.gz Juv4_2.fastq.gz  Juvenile Juvenile_Rep5 Juv1_5.fastq.gz Juv5_2.fastq.gz  Muscle Muscle_rep1 Mus1_1.fastq.gz Mus1_2.fastq.gz  Muscle Muscle_rep2 Mus2_1.fastq.gz Mus2_1.fastq.gz  Kidney Kidney_rep1 Kid1_1.fastq.gz Kid1_2.fastq.gz  Kidney Kidney_rep2 Kid2_1.fastq.gz Kid2_2.fastq.gz  Liver Liver_rep1 Liv1_1.fastq.gz Liv1_2.fastq.gz  Liver Liver_rep2 Liv2_1.fastq.gz Liv2_1.fastq.gz  perl trinityrnaseq-v2.15.1/util/ filter_low_expr_transcripts.pl --matrix raw_counts.matrix --transcripts trinity_anchovy.Trinity.fasta -highest_iso_only --gene_to_trans_map trinity_anchovy.Trinity.fasta.gene_trans_map  #Differential_Expression_Analysis_with_DeSeq2  #meta data for DESeq2  sample tissue condition platform  Caudal_rep1 Caudal Other 2  Caudal_rep2 Caudal Other 2  Caudal_rep3 Caudal Other 2  Fin_rep1 Fin Other 2  Fin_rep2 Fin Other 2  Fin_rep3 Fin Other 2  Gill_rep1 Gill Other 2  Gill_rep2 Gill Other 2  Gill_rep3 Gill Other 2  Ovary_rep1 Ovary Ovary 1  Ovary_rep2 Ovary Ovary 1  Ovary_rep3 Ovary Ovary 1  Testis_rep1 Testis Testis 1  Testis_rep2 Testis Testis 1  Testis_rep3 Testis Testis 1  Juvenile_Rep1 Juvenile Other 1  Juvenile_Rep2 Juvenile Other 1  Juvenile_Rep3 Juvenile Other 1  Juvenile_Rep4 Juvenile Other 1  Juvenile_Rep5 Juvenile Other 1  Muscle_rep1 Muscle Other 1  Muscle_rep2 Muscle Other 1  Kidney_rep1 Kidney Other 1  Kidney_rep2 Kidney Other 1  Liver_rep1 Liver Other 1  Liver_rep2 Liver Other 1  dds <- DESeqDataSetFromMatrix(countData=counts,  colData=meta,  design=~condition + platform, tidy = TRUE)  dds <- DESeq(dds)  res <- results(dds)  ovary_vs_other_tissues_DE_results = results(dds, contrast=c("condition","Ovary","Other"))  testis_vs_other_tissues_DE_results = results(dds, contrast=c("condition","Testis","Other")) |
| --- |

***#Sequence Annotations***

| TransDecoder.LongOrfs –t trinity_anchovy.Trinity.fasta  hmmscan --cpu 28 --domtblout pfam.anchovy.domtblout Pfam-A.hmm longest_orfs.pep  diamond blastp -d uniref90 -q longest_orfs.pep --sensitive -o blastp.anchovy.outfmt6 -f 6 -e 0.00001 --top 5 -p 28  TransDecoder.Predict -t trinity_anchovy.Trinity.fasta --retain_pfam_hits pfam.anchovy.domtblout --retain_blastp_hits blastp.anchovy.outfmt6  tmhmm –short trinity_anchovy.Trinity.fasta.transdecoder.pep > anchovy.tmhmm.out  signalp -f short –n anchovy.signalp.out trinity_anchovy.Trinity.fasta.transdecoder.pep  RnammerTranscriptome.pl –transcriptome trinity_anchovy.Trinity.fasta --path_to_rnammer /rnammer_v1.2/rnammer  TRAPID Online Tool [http://bioinformatics.psb.ugent.be/trapid_02/]  EggNOG Online Tool [http://eggnog5.embl.de/#/app/home]  ShinyGO Online Tool [http://bioinformatics.sdstate.edu/go/]  STRING Online Tool [https://string-db.org/] |
| --- |

***#Positive_Selection_Analysis***

| perl gmst.pl trinity_anchovy.Trinity.fasta  # Reciprocal Best Hits (RBH)  #database_anchovy+query_danio  makeblastdb -in trinity_anchovy.Trinity.fasta.transdecoder.pep -title trinity_anchovy.Trinity -dbtype prot -out trinity_anchovy.Trinity -parse_seqids  blastp –query Danio_rerio.GRCz11.pep.all.fa –db trinity_anchovy.Trinity –out anchovy_vs_Danio.txt -num_alignments 1 -evalue 1e-30  #database_danio+query_anchovy  makeblastdb –in Danio_rerio.GRCz11.pep.all.fa –title Danio_rerio.GRCz11.pep -dbtype prot –out Danio_rerio.GRCz11 -parse_seqids  blastp –query trinity_anchovy.Trinity.fasta.transdecoder.pep –db Danio_rerio.GRCz11.pep –out Danio_vs_anchovy.txt -num_alignments 1 -evalue 1e-30  jvenn Online Tool [https://jvenn.toulouse.inrae.fr/app/index.html] used for extracting common hits.  Ensembl BioMart Online Tool [http://www.ensembl.org/biomart/] was used to extract single copy orthologous genes (CDS) for remaining fish species.  #Prank_and_Guidance  perl guidance.pl --seqFile ortho.fasta --msaProgram PRANK --seqType codon --outDir guidance_output  #PhyML  phyml -i alignment_peptide.fasta -d aa  Paml_CodeML  codeml codeml.ctl  #CodeML_control_file  seqfile = $ortho1.phy  treefile = PhyML.tree  outfile = $ortho1_output  noisy = 3  verbose = 0  runmode = 0  model = 2  Nsites = 2  icode = 0  fix_kappa = 0  kappa = 2.5  fix_omega = 1  omega = 1  getSE = 0 |
| --- |
