# Supplementary material for: Global expression pattern of genes containing positively selected sites in European anchovy (Engraulis encrasicolus L.) may shed light on teleost reproduction
Source: PLoS One. 2023 Aug 11;18(8):e0289940. doi: 10.1371/journal.pone.0289940 (PMC10420382; doi:10.1371/journal.pone.0289940)
Supplement: S3 File — (DOCX) [file pone.0289940.s010.docx]

**Supplementary File 3.** The assessment of cross-platform variation between Illumina HiSeq2000 and MGI DNBSEQ-400 platforms regarding base quality, read lengths, sequencing depth, and sample clustering.

In this study, a subset of tissue samples, including ovary, testis, juvenile, kidney, liver, and muscle, were subjected to sequencing using the Illumina HiSeq2000 platform. In contrast, gill, cauda, and fin tissues were sequenced using the MGI DNB-SEQ 400 platform. This supplementary data is intended to demonstrate that cross-platform variation is not pronounced when considering factors such as base quality, read lengths, sequencing depth, and sample clustering.

The read lengths, error rates, and sequencing depths of platforms can lead to variations in the quality and quantity of the reads generated, potentially introducing biases in the gene expression analysis. The studies showed that MGI and Illumina are similar technologies, so the effects may not be too strong. For example;

**1)** Comparison between MGI and Illumina sequencing platforms for whole genome sequencing (the most of comparison studies focused on genome or exome sequencing).

Kim et al. (2021) showed that “*Overall, MGI and Illumina sequencing platforms showed comparable levels of sequencing quality, uniformity of coverage, percent GC coverage, and variant accuracy; thus we conclude that the MGI platforms can be used for a wide range of genomics research fields at a lower cost than the Illumina platforms.*”

Jeon et al. (2021) showed that “*the performances of the MGISEQ-2000 and DNBSEQ series were similar to those of Illumina NGS systems. Overall, we concluded that the three platforms (NovaSeq 6000, MGISEQ-2000, and DNBSEQ-T7) are highly concordant, and that MGISEQ-2000 and DNGSEQ-T7 can be fully compatible alternatives to NovaSeq 6000 in WGS analysis.*”

**2)** Comparison of the MGISEQ-2000 and Illumina HiSeq 4000 sequencing platforms for RNA sequencing

Jeon et al. (2019) showed that “*the MGISEQ-2000 produced high-quality sequence data comparable to the data obtained by the HiSeq 4000, at half the price. We suggest that the MGISEQ-2000 is a promising sequencing platform for whole-transcriptomics studies with high performance and low cost.*”

These studies emphasized that the MGI and Illumina platforms are significantly similar regarding read length, base quality, and sequence depth. When we examine the data set in our study;

- read length is similar between Illumina (101 nt) and MGI platform (100 nt) (please see Supplementary Table 1 (**Table S1**) for further details.
- When we examine the Phred score of the reads obtained from both platforms, we observe that the score (*Q*) is mostly greater than 30 (**Figure 1A** and **1B**).
- To investigate the effect of the platforms used, we applied a dimensionality reduction technique (PCA) to determine whether the samples cluster by platforms or by tissue types. As seen in **Figure 2**, the samples were not clustered based on the platform.

**References:**

Jeon, S. A., Park, J. L., Kim, J. H., Kim, J. H., Kim, Y. S., Kim, J. C., & Kim, S. Y. (2019). Comparison of the MGISEQ-2000 and Illumina HiSeq 4000 sequencing platforms for RNA sequencing. Genomics & informatics, 17(3).

Jeon, S. A., Park, J. L., Park, S. J., Kim, J. H., Goh, S. H., Han, J. Y., & Kim, S. Y. (2021). Comparison between MGI and Illumina sequencing platforms for whole genome sequencing. Genes & Genomics, 43, 713-724.

Kim, H. M., Jeon, S., Chung, O., Jun, J. H., Kim, H. S., Blazyte, A., .& Bhak, J. (2021). Comparative analysis of 7 short-read sequencing platforms using the Korean Reference Genome: MGI and Illumina sequencing benchmark for whole-genome sequencing. GigaScience, 10(3), giab014.


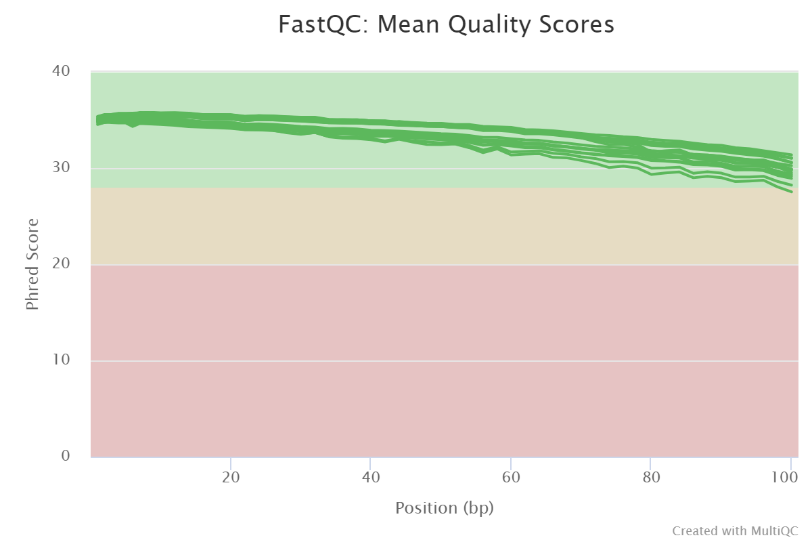


**Figure 1A.** The sequence quality (per base) histogram of reads generated by DNBSEQ-400 instrument (MGI).


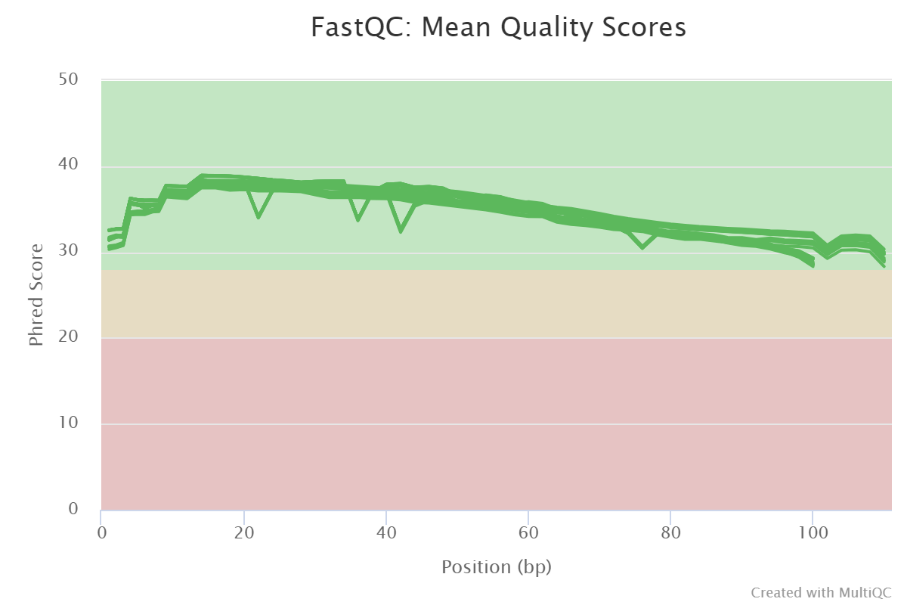


**Figure 1B.** The sequence quality (per base) histogram of reads generated by HiSeq2000 instrument (Illumina).


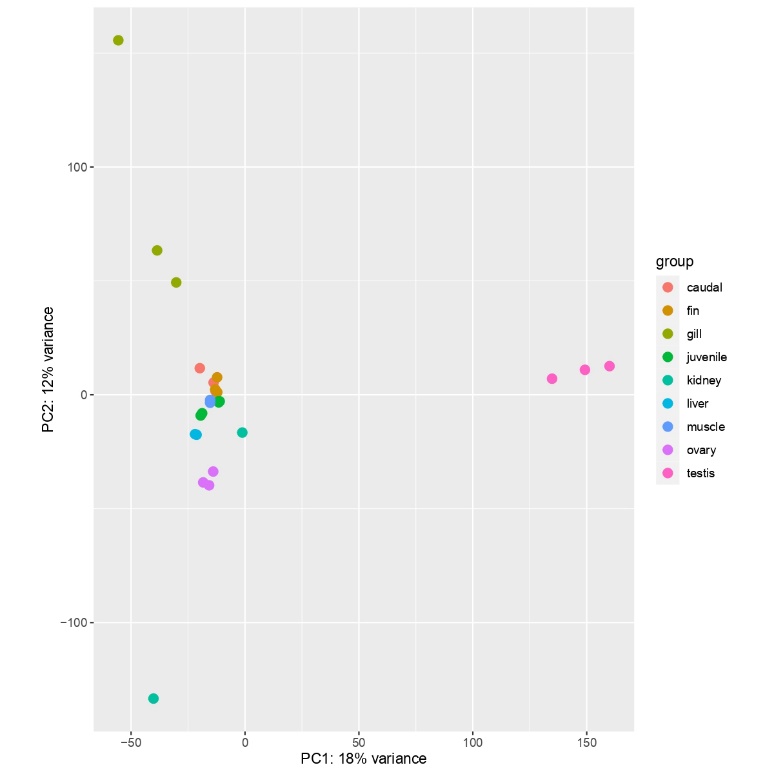


Şekil 2. The PCA plot of tissue samples.
